# Dosiomics: Extracting 3D Spatial Features From Dose Distribution to Predict Incidence of Radiation Pneumonitis

**DOI:** 10.3389/fonc.2019.00269

**Published:** 2019-04-12

**Authors:** Bin Liang, Hui Yan, Yuan Tian, Xinyuan Chen, Lingling Yan, Tao Zhang, Zongmei Zhou, Lvhua Wang, Jianrong Dai

**Affiliations:** Department of Radiation Oncology, National Cancer Center, National Clinical Research Center for Cancer, Cancer Hospital, Chinese Academy of Medical Sciences and Peking Union Medical College, Beijing, China

**Keywords:** dosiomics, radiomics, dose distribution, pneumonitis prediction, logistic regression

## Abstract

Radiation pneumonitis (RP) is one of the major toxicities of thoracic radiation therapy. RP incidence has been proven to be closely associated with the dosimetric factors and normal tissue control possibility (NTCP) factors. However, because these factors only utilize limited information of the dose distribution, the prediction abilities of these factors are modest. We adopted the dosiomics method for RP prediction. The dosiomics method first extracts spatial features of the dose distribution within ipsilateral, contralateral, and total lungs, and then uses these extracted features to construct prediction model via univariate and multivariate logistic regression (LR). The dosiomics method is validated using 70 non-small cell lung cancer (NSCLC) patients treated with volumetric modulated arc therapy (VMAT) radiotherapy. Dosimetric and NTCP factors based prediction models are also constructed to compare with the dosiomics features based prediction model. For the dosimetric, NTCP and dosiomics factors/features, the most significant single factors/features are the mean dose, parallel/serial (PS) NTCP and gray level co-occurrence matrix (GLCM) contrast of ipsilateral lung, respectively. And the area under curve (AUC) of univariate LR is 0.665, 0.710 and 0.709, respectively. The second significant factors are V_5_ of contralateral lung, equivalent uniform dose (EUD) derived from PS NTCP of contralateral lung and the low gray level run emphasis of gray level run length matrix (GLRLM) of total lungs. The AUC of multivariate LR is improved to 0.676, 0.744, and 0.782, respectively. The results demonstrate that the univariate LR of dosiomics features has approximate predictive ability with NTCP factors, and the multivariate LR outperforms both the dosimetric and NTCP factors. In conclusion, the spatial features of dose distribution extracted by the dosiomics method effectively improves the prediction ability.

## Introduction

Radiation pneumonitis (RP) is one of the major toxicities of thoracic radiation therapy. The clinical symptoms range from fever, cough to pulmonary function failure, which may occur during the first 6 months after irradiation. Reducing the prescription dose could lower the risk of RP incidence, but also impairs tumor control. An accurate RP predictor (or prediction model) is desired to “safely” irradiate the tumor target without increasing the risk of RP incidence.

RP incidence is directly associated with the dose distribution within lung volume. Dosimetric factors, such as mean lung dose (MLD) and the lung volume within which the dose is greater than xGy (V_x_), are widely used for RP prediction. Boonyawan et al. reported that RP incidence increases with V_10_ and V_20_ (1). Ramella et al. found that RP incidence is associated with V_20_ and V_30_ (2). Briere et al. reported that RP incidence significantly increases if the sparing lung volume is <1852cc (receiving dose≤40Gy) (3). Pinnix et al. reported that V_5_ has better prediction capability than V_10_, V_15_, and V_20_ (4). Palma et al. analyzed 836 patient cases from international institutions and concluded that symptomatic RP is associated with V_20_, and fatal RP associated with the mean dose per day during treatment (5). Those studies demonstrate that the dosimetric factors are associated with RP incidence. Although enlightening for understanding the causes of RP incidence, the conclusion of those studies differs from individual institution or dataset.

The dosimetric factors only utilize partial information contained in the dose distribution. For instance, V_x_ is only a discrete point on the dose volume histogram (DVH) curve. Compared with dosimetric factors, the normal tissue complication probability (NTCP) model utilizes all information of the DVH curve by compressing the entire curve to a single factor with dose response functions (DRFs). Better prediction performance could be achieved by fitting the DRF parameters (6–8). And the standard deviation of the fitted parameters is 16.5% between different institutions (9). The improved prediction ability of NTCP factors are partly contributed by the introduction of DRF, but more importantly because the NTCP factors utilize all information of DVH curve. However, since the DVH curve does not take the spatial information into consideration, completely different dose distributions may result in identical DVH curve, and then identical NTCP factors.

By investigating the prediction ability of dosimetric and NTCP factors, it is reasonable to hypothesize that if the spatial information of dose distribution were utilized properly, the prediction ability should be further improved. The recently emerged radiomics method extracts numerous spatial features from medical images and uses these features to predict therapeutic responses (10–12). Enlightened by those works, the “**dosiomics**” method has been proposed, which attempts to extract the spatial features from dose distribution for radiotherapy response prediction (13–15). In this work, we adopted the dosiomics method for RP incidence prediction. The prediction ability of the dosiomics features is validated using 70 non-small cell lung cancer (NSCLC) patients treated with volumetric modulated arc therapy (VMAT) radiotherapy, and further evaluated by comparing with the dosimetric and NTCP factors.

## Methods and Materials

### Patient Data

Seventy NSCLC patients treated in our institution from 2013 to 2016 are used in this study. All patients were treated with 6MV VMAT without surgical operation. Treatment plans were designed using Pinnacle treatment planning system (v 9.0). The slice spacing of planning CT image was 5 mm, and the grid spacing of dose calculation was 4 × 4 × 4 mm. The dose was prescribed to 95% of the planning target volume (PTV). RP was graded from 0 to 5 according to Common Terminology Criteria for Adverse Events (CTCAE v3.0). Bootstrap method is adopted to address the issue of limited dataset. Bootstrap of original dataset is performed 1,000 times, and the resulting 1,000 bootstrap samples are used as training datasets for both the univariate and multivariate LR.

### Feature Extraction

The dosiomics features are extracted from the dose distribution within ipsilateral, contralateral and total lungs, separately. The extracted features are a set of indices, such as autocorrelation, sum of squares and cluster prominence etc, derived from the gray level co-occurrence matrix (GLCM) and gray level run length matrix (GLRLM). The calculation formulas can be referred in (16). All the extracted features are normalized to zero mean and unit variance with z-score normalization before further processing.

### Univariate Analysis

The endpoint of this study is the occurrence of grade ≥2 RP. Prediction model is built using LR, of which the coefficient is derived using maximum likelihood estimation method. For each extracted feature, univariate LR is performed 1,000 times based on the bootstrap samples of original dataset. The mean AUC of training datasets (bootstrap samples) is calculated. The most significant feature is determined as the feature with maximal mean training AUC. The final coefficient is determined as the median of the 1,000 resulting coefficients. The predictive ability of each single feature is evaluated on the original entire dataset by the area under curve (AUC) of receiver operating characteristic (ROC) (17).

### Multivariate Analysis

According to the one tenth rule, the number of predictors of multivariate LR should be one tenth of the outcome events. Recent studies show that the one tenth rule is generally too conservative, and can be relaxed for the logistic and Cox regression (18). The outcome event (grade ≥2 RP occurrence) of this study is 15, and the number of predictors of multivariate LR is relaxed to 2.

In this study, all possible two-feature combinations are traversed to search for the optimal combination. For each combination, multivariate LR and Spearman test between the two features are performed 1,000 times using bootstrap samples. The optimal combination is determined as the combination with maximal mean training AUC while the mean Spearman correlation within [−0.8, 0.8]. The Spearman correlation threshold excludes the combination of strongly correlated features to prevent overfitting. The final coefficient is also determined as the median of the 1,000 resulting coefficients, and the multivariate LR model is validated on the entire dataset.

### Dosimetric and NTCP Factors

The predictive ability of dosiomics features is further evaluated by comparing with the dosimetric and NTCP factors. Both univariate and multivariate analyses are performed using: 1. dosimetric factors and 2. NTCP factors. As listed in Table 1, the dosimetric factors include V_5_, V_10_, V_15_, V_20_, and MLD. The NTCP factors are two sets of equivalent uniform dose (EUD) and NTCP factors of Lyman (19) and parallel/serial (PS) models (20). All factors are calculated for the ipsilateral, contralateral and total lungs, separately.

**Table 1:** Dosimetric and NTCP factors.

Dosimetric factors	V_5_, V_10_, V_15_,V_20_ and MLD
NTCP factors	EUD_L_, NTCP_L_, EUD_PS_ and NTCP_PS_

With DVH reduction technique, the heterogeneous dose distribution is reduced to a single EUD_L_ parameter, which by definition has the same complication probability with the original heterogeneous dose distribution. Lyman model assumes that all sub-volumes are of the same contribution to side effects, and the NTCP factor (NTCP_L_) is calculated as:

(1) $$\begin{array}{rcl} \text{NTC}{\text{P}}_{\text{L}} & = & \frac{1}{\sqrt{2\pi }}\overset{T}{\underset{-\infty }{\int }}\exp\left(-\frac{t^{2}}{2}\right)dt \end{array}$$

(2) $$\begin{array}{rcl} T & = & \frac{EUD-TD_{50}}{mTD_{50}}, \end{array}$$

$$\begin{array}{rcl} EUD_{L} & = & {\left(\frac{1}{N}\underset{i}{\sum }D_{i}^{a}\right)}^{\frac{1}{a}} \end{array}$$

where *TD*_50_ is the tolerance dose, under which the complication probability of normal tissue is 50%. *m* is the curve slope at *TD*_50_.

The PS model considers the normal tissues are composed of a large number of microscopic functional sub-volumes. The parallel model supposes the sub-volumes are independent. Side effects occur only if a significant number of sub-volumes are destroyed, and small portion of the damaged sub-volumes will not cause side effects. In contrast, the serial model assumes the sub-volumes are dependent. Side effects occur even only a small portion of sub-volumes are damaged. The entire volume is an arbitrary mixture of both serial and parallel sub-volumes. The NTCP (NTCP_PS_) factor is calculated as:

(3) $${\text{NTCP}}_{\text{PS}}=(1-\prod_{i=1}^{N}{\left(1-P{\left(D_{i}\right)}^{k}\right)}^{N}{)}^{\frac{1}{k}},$$

$$\begin{array}{rcl} P\left(D\right) & = & 2^{-\exp\left(e\cdot m\left(1-\frac{D}{TD_{50}}\right)\right)} \end{array}$$

where *k* is the ratio of serial to parallel sub-volume. Like Lyman model, *m* is the slope of NTCP curve at *TD*_50_. EUD_PS_ is calculated according to formula proposed in (21):

(4) $$\begin{array}{r} \text{EU}{\text{D}}_{\text{PS}}=TD_{50}\frac{e\;\cdot \;m-\ln\;\left(-\ln\;\left(\text{NTC}{\text{P}}_{\text{PS}}\right)\right)}{e\;\cdot \;m-\ln\;\left(\ln\;\left(2\right)\right)} \end{array}$$

The study presented in (22) optimized the parameters of NTCP models for grade≥2 RP prediction based on 382 thoracic patient cases. As the endpoint is the same and patient population is similar with our study, we directly used the same parameters, which are listed in Table 2.

**Table 2:** NTCP model parameters.

	**TD_**50**_**	***a* or *k***	***m***
Lyman	30.8	0.99	0.37
PS	34.0	0.06	0.90

*The 3rd column lists the value of a for Lyman model and k or PS models*.

The dose distribution within lung volumes is derived from the treatment plan data in DicomRT format. This procedure is implemented with Matlab software (MathWorks, Natick, MA). Feature extraction is implemented with the python pyradiomics package (v2.0.0) (16). LR is implemented in R language, with the stats (v3.4.1) package (23).

## Results

**Figure 1:**
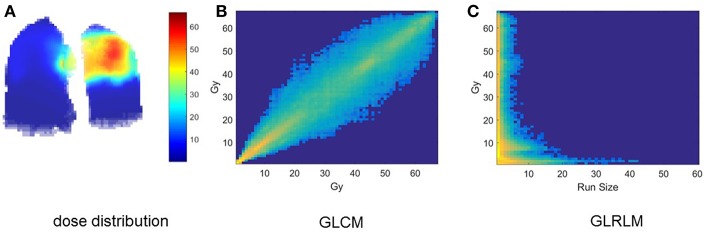
Intermediate results of dosiomics method. **(A)** 3D dose distribution, **(B)** GLCM, and **(C)** GLRLM of total lung.

Clinical factors of analyzed patients are listed in Table 3. Figure 1A shows the dose distribution within total lungs, and Figures 1B,C shows the corresponding GLCM and GLRLM in logarithmic scale for the sake of clearance. Twenty and sixteen features, which are commonly used in radiomics studies, are derived from GLCM and GLRLM, respectively. All the features are calculated for the dose distribution within ipsilateral, contralateral and total lungs, separately. In total, 129 [(27 + 16) × 3] features are extracted for each patient.

**Table 3:** Clinical factors.

**Characteristic**	**Value**
**STAGE**	
I	4 (5.7%)
II	5 (7.1%)
III	53 (75.7%)
IV	8 (11.4%)
**SEX**	
Male	61 (87.1%)
Female	9 (12.9%)
**AGE**	
Range	35-84
Mean ± Std.	61 ± 10
**TUMOR LOCATION**	
Left	33 (47.1%)
Right	37 (52.9%)
**KPS**	
≤80	41 (58.6%)
>80	29 (41.4%)
**CONCURRENT CHEMOTHERAPY**	
Yes	38 (54.3%)
No	32 (45.7%)
**SMOKING HISTORY**	
Yes	60 (85.7%)
No	10 (14.3%)
**PRESCRIPTION DOSE (Gy)**	
Single fraction	2.27 ± 0.85
Total	59.10 ± 5.67
**RP GRADE**	
≥2	15 (21.4%)
<2	55 (78.6%)

*KPS, Karnofsky performance status*.

### Univariate Analysis

Table 4 lists the results of univariate analysis. The most significant predictors of the three sets of factors/features are the MLD (MLD^I^), NTCP_PS_ (${\text{NTCP}}_{\text{PS}}^{\text{I}}$) and GLCM (GLCM^I^) contrast of ipsilateral lung, respectively. The GLCM contrast measures local variation of dose distribution. It is interesting to notice that the most significant predictors are all derived from the dose distribution within ipsilateral lung. The median and 10–90th% odds ratio (OR) of the most significant factor/feature and constant term are listed in Table 4. The OR can be interpreted as: increasing the corresponding factor by one unit the probability of RP incidence increases by *OR* times. Increasing MLD^I^, ${\text{NTCP}}_{\text{PS}}^{\text{I}}$ and GLCM^I^ contrast by one unit will increase the probability of RP incidence by 1.667, 2.041 and 2.010 times. The range of 10–90th% OR measures the repeatability of the derived predictive model. The 10–90th% OR range of GLCM^I^ contrast is greater than MLD^I^ but lower than ${\text{NTCP}}_{\text{PS}}^{\text{I}}$. The OR of constant term is the ratio of RP probability over non-RP probability of the dataset without using any predictor. For the three LR models, the median and 10–90th% ORs of constant term are almost identical. The AUC values of NTCP and dosiomics factor/feature are approximate, but higher than dosimetric factors.

**Table 4:** Univariate analysis results.

	**Feature/factor**	**Median OR**	**10th−90th% OR**	**AUC**
Dosimetric	MLD^I^	1.667	1.037–2.842	0.665
	Constant	0.256	0.210–0.275	
NTCP	${\text{NTCP}}_{\text{PS}}^{\text{I}}$	2.041	1.332–4.119	0.710
	Constant	0.246	0.206–0.271	
Dosiomics	GLCM^I^ contrast	2.010	1.383–3.772	0.709
	Constant	0.255	0.219–0.282	

*The superscript “I” in the second column indicates the feature is extracted from the dose distribution of ipsilateral lung*.

### Multivariate Analysis

**Figure 2:**
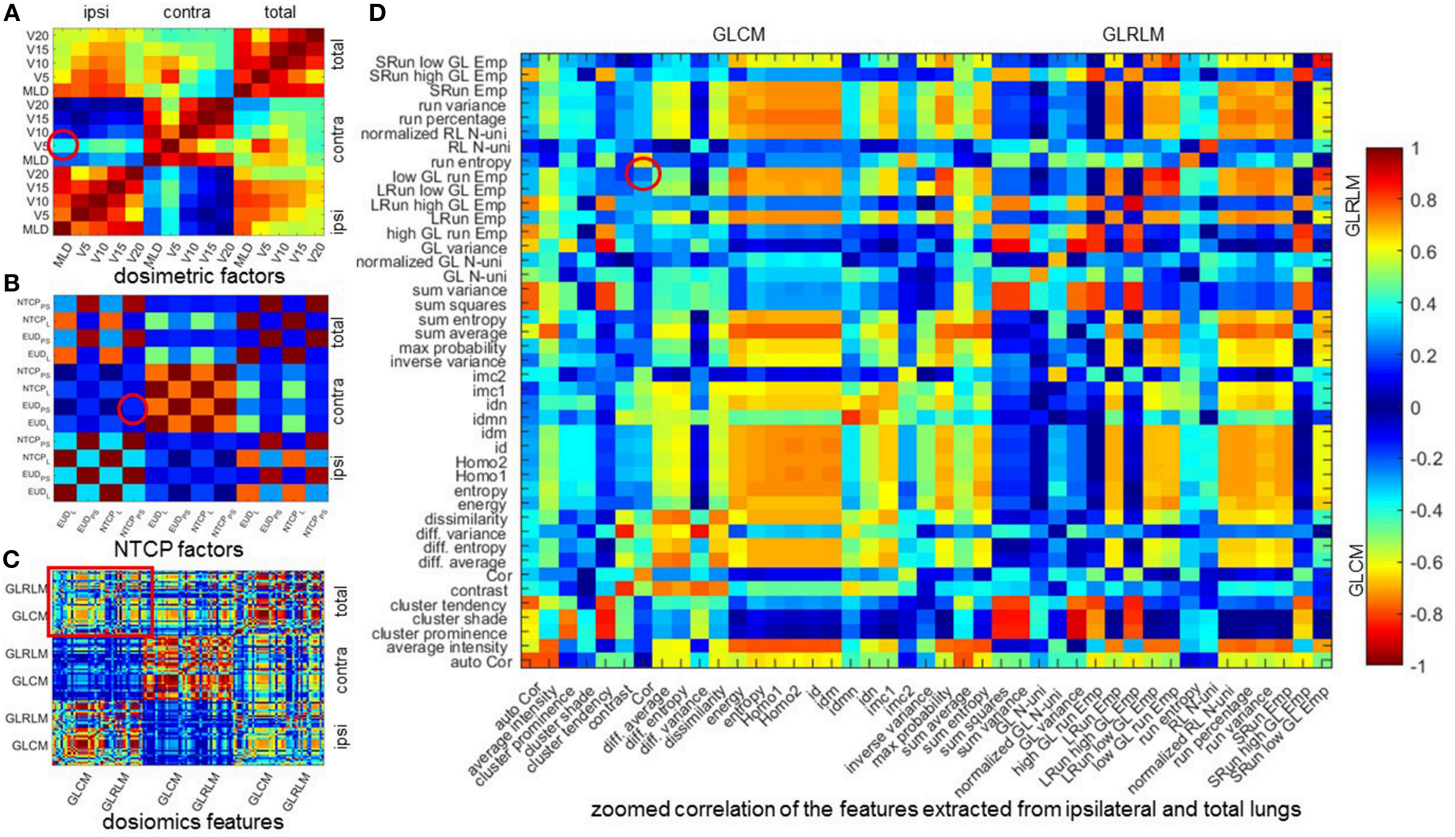
Mean Spearman correlation of 1,000 bootstrap samples. **(A–C)** mean Spearman correlation of dosimetric factors, NTCP factors, and dosiomics features. **(D)** zoomed correlation of the features extracted from ipsilateral and total lungs. All the 4 figures are diagonal symmetric. For **(A–C)**, the features are sorted in the order of ipsilateral, contralateral, and total lungs from left to right and from top to down. In **(D)**, the features are sorted the order of GLCM and GLRLM. The correlation of the features of the optimal combination are denoted with red circle. Cor, correlation; Emp, emphasis; GL, gray level; Homo, homogeneity; LRun, long run; SRun, short run; N-uni, non-uniformity; RL, run length.

Table 5 lists the results of multivariate analysis. The optimal combinations all contain the most significant single predictors. The second significant predictors are V_5_ of contralateral lung (${\text{V}}_{5}^{C}$), EUD_PS_ (${\text{EUD}}_{\text{PS}}^{C}$) of contralateral lung and GLRLM (GLRLM^T^) low gray level run emphasis of total lungs, respectively. ${\text{V}}_{5}^{C}$ represents the lower dose within contralateral lung. ${\text{EUD}}_{\text{PS}}^{C}$ by definition is the uniform dose that has the same complication probability with the original heterogeneous dose distribution. The GLRLM^I^ low gray level run emphasis measures the region of low dose, with a higher value indicating a greater concentration of low dose distribution. The most significant single factors/features are all extracted from ipsilateral lung, while the second from either contralateral or total lungs. As shown in Figure 2, the factors/features extracted from same dose distributions are more correlated, while the factors/features extracted from different dose distributions are less correlated, especially the factors/features of ipsilateral and contralateral lungs. In order to prevent overfitting, the strongly correlated factors/features are excluded. This explains why the second predictors are derived from either contralateral or total lungs.

**Table 5:** Multivariate analysis results.

	**Feature/factor**	**Median OR**	**10th−90th% OR**	**Spearman correlation**	**AUC**
Dosimetric	MLD^I^	1.530	0.879–2.599	0.378 ± 0.110	0.676
	${\text{V}}_{5}^{C}$	1.360	0.860–2.264		
	Constant	0.241	0.184–0.272		
NTCP	${\text{NTCP}}_{\text{PS}}^{\text{I}}$	1.996	1.311–4.938	−0.176 ± 0.129	0.744
	${\text{EUD}}_{\text{PS}}^{C}$	1.183	0.772–1.931		
	Constant	0.240	0.195–0.268		
Dosiomics	GLCM^I^ contrast	1.843	1.276–3.519	−0.168 ± 0.087	0.782
	GLRLM^T^ low GL run emphasis	1.232	1.028–1.601		
	Constant	0.211	0.114–0.261		

*The superscript “I” in the second column indicates the feature is extracted from the dose distribution of ipsilateral lung, “C” and “T” for contralateral and total lung, respectively*.

The mean Spearman correlation between the two selected factors/features of 1,000 bootstrap samples and its standard deviation are also listed in Table 5. MLD^I^ and ${\text{V}}_{5}^{C}$ is positive correlated. This is because increasing the MLD of ipsilateral lung would increase the scatter dose delivered to contralateral lung thus increase the value of V_5_. For NTCP and dosiomics factors/features, the Spearman correlation is negative and of lower magnitude, indicating that the selected predictors are weakly negative correlated.

For dosiomics features, the increase of AUC is obvious when switching from univariate LR to multivariate LR. On the other hand, the increase of AUC for dosimetric and NTCP factors is limited. This is because either the dosimetric or NTCP factors describe the dose distribution from the similar perspective. Adding another predictor will not significantly improve the predictive ability. On the contrary, the dosiomics features display a rich diversity, which is benefit for revealing the hidden correlation with RP incidence.

## Discussion

We investigated the published studies on the correlation between dosimetric factors and RP incidence, and found the conclusions differ from individual institution or dataset. The quantitative analysis of normal tissue effects in the clinic (QUANTEC) summarized available published data and performed a logistic regression between MLD and RP (9). Despite the differences in patient selection and RP grade of published data, an obvious trend could be observed: the probability of RP incidence increases with MLD. This conclusion supports our finding: MLD^I^ is the most significant dosimetric predictor. Most published studies on the correlation of NTCP factors and RP incidence focus on fitting the parameters of NTCP models to better predict RP incidence. In this study, we directly used the optimized parameters presented in (22), and found that ${\text{NTCP}}_{PS}^{\text{I}}$ is the most significant predictor. Tsougos et al. (7) also reported that PS model outperforms the rest NTCP models for RP (grade 2) prediction of breast cancer radiotherapy. Both studies demonstrate that RP occurs if significant sub-volumes are damaged. This conclusion is further validated by the study reported in (3), which found RP incidence significantly increases if the sparing lung volume (dose ≤ 40Gy) is less than 1852cc.

The results of multivariate LR demonstrate that the prediction ability of dosiomics features outperform dosimetric and NTCP factors. Meanwhile the NTCP factors has better performance than the dosimetric factors. The results validate the hypothesis that the predictive ability improves with more information of the dose distribution are used by the prediction model. The application of dosiomics method is not limited to RP prediction. It is suitable for any radiotherapy outcome, either positive (like survival, control rate) or negative (like normal tissue damage, complication).

We have to admit that the patient dataset of this study is limited. In order to address this issue, the bootstrap approach is adopted. The coefficients of univariate and multivariate LR is determined as the median of the fitting results using bootstrap samples. The predictor number of multivariate LR is set to 2 to avoid overfitting. For dosiomics features, the improvement on predictive ability is significant when switching from univariate LR to multivariate LR. With the diverse dosiomics features, it is reasonable to assume that the predictive ability could be further improved when the patient dataset is enlarged with more positive cases, in which case more predictors could be included in multivariate LR.

Except for enlarging the patient dataset, another promising direction to further improve the predictive ability is to dig deeper of the patient dataset. Dose distribution, even strongly correlated with RP incidence, is not the unique factor. Other clinical factors, such as the age, smoking history, chemotherapy, are also found to be associated with RP incidence. In addition, other “omics” features, such as the features extracted from radiomics, genome and proteomics, also provide insightful information. All these factors/features could be integrated into the model to improve its prediction ability and robustness.

Although the dosiomics features demonstrate good prediction ability, the understanding of these features is still qualitative. The main reason is the process of transform dose distribution into GLCM and GLRLM cannot be accurately described with analytic function. Therefore the features based on GLCM and GLRLM are not as simple and straightforward as dosimetric factors. Based on the finding of this study, we qualitatively conclude that the higher local dose variation within ipsilateral lung and the greater low dose region of total lungs, the greater probability of RP incidence. Furthermore, how to utilize the features for treatment plan design is not quite clear. In other words, currently the dosiomics method is limited to RP incidence prediction. How to use the dosiomics method to prevent RP incidence during treatment planning is one goal of our future study.

## Ethics Statement

This study was carried out in accordance with the declaration of Helsinki and approved with exemption from informed consent by the independent ethics committee of cancer hospital, Chinese academy of medical sciences (No. NCC2015 G-15).

## Author Contributions

BL, HY, and JD conceived the project and wrote the paper. XC, YT, and LY collected and analyzed the data. TZ, ZZ, and LW provided expert clinical knowledge. All authors edited the manuscript.

### Conflict of Interest Statement

The authors declare that the research was conducted in the absence of any commercial or financial relationships that could be construed as a potential conflict of interest.
